# The role of natural experiments in hepatology research: filling the gap between clinical trials and service evaluations

**DOI:** 10.1097/HC9.0000000000000121

**Published:** 2023-04-14

**Authors:** Ryan M. Buchanan, Alex Smith, Ian Rowe

**Affiliations:** 1 Faculty of Medicine, University of Southampton, Southampton, UK; 2 National Institute for Health and Care Research, Southampton Biomedical Research Centre, University Hospital Southampton, Southampton, UK; 3 Southampton Hepatology Department, University Hospital Southampton, Southampton, UK; 4 Leeds Institute for Medical Research, University of Leeds, Leeds, UK; 5 Leeds Liver Unit, St James’s University Hospital, Leeds, UK

## Abstract

Research developing and testing interventions that address the social determinants of liver disease are urgently needed; however, this cannot be achieved using conventional clinical research designs. A different approach is needed to conduct widely applicable, inclusive, and community-based research that addresses upstream factors driving liver morbidity. Natural experimental studies encompass a well-established field of research methodology that is less familiar to clinical hepatologists than conventional research methods such as the randomized control trial. The key strength of natural experiments is that, when robustly designed, they can be used to imply causality from routinely collected data. As such, they are well placed to test the impact of community interventions that aim to address social determinants of liver disease that cannot feasibly be assessed in a randomized control trial. In this review, we define natural experiments and their potential utility. We then work through examples of where they have already been used in clinical hepatology to highlight a range of research designs, analytical approaches, and best practices regarding their conduct and reporting. In doing so, we hope to equip clinical hepatologists with another tool to ensure the hepatology community can meet the global liver disease epidemic with evidence-based interventions.

## INTRODUCTION

The European Association for the Study of the Liver–Lancet commission stresses the inconsistency in models of care for liver disease in Europe and the scarcity of programs delivering testing and treatment for early-stage disease. The commission highlights the enormous number of lives that could be saved if measures that address disease prevention and detection are properly validated and implemented.^1^

Both the European Association for the Study of the Liver–Lancet commission and field leaders in the US^2^ emphasize the need to study the “social determinants of liver disease” (eg, stigma, discrimination, and asymmetrical resources allocation^3^) if meaningful progress is to be made. Presently, the quantity and quality of interventional studies addressing upstream social determinants of health in gastroenterology and hepatology are described as “grim.”^4^ There are many barriers to conducting research in this area: (1) the causal relationship between social determinants of health and liver disease is convoluted and complex, (2) in the short term, intervention leads to “soft” nonclinical outcomes (eg, reduced alcohol intake), (3) interventions are often multimorbidity focused, and (4) potential research participants are predominantly in the community rather than hospital settings—limiting the accessibility of the research population to predominantly hospital-based hepatologists.^3^ An important additional contributory factor to this lack of evidence is our collective professional insistence on using clinical research methods to solve what are essentially public health problems. This leads to a lack of diversity in research^5^ and a particular lack of evidence for interventions targeting social determinants of liver health in marginalized and deprived populations—a lack of evidence that leads to a lack of spending and policy change.^3^,^6^

The gold standard clinical experiment is the randomized controlled trial (RCT). An RCT has 4 defining features: (1) it includes 2 or more groups, (2) 1 or more group is assigned to a treatment or series of treatments, (3) subjects are randomly assigned to 1 group, and (4) the treatment can be manipulated by the researcher.^7^ The random assignment of the individuals to groups means that “on average,” they should have the same characteristics. Thus, statistically similar groups are exposed at the same time to 2 or more different conditions, which reduces or eliminates confounding and supports causal inferences. There are, however, many circumstances when an RCT is impossible and many cases when, even though an RCT is possible, such a trial has not been funded, has not been done and will not be done in a timescale that helps the policy maker or clinician.

**FIGURE 1:**
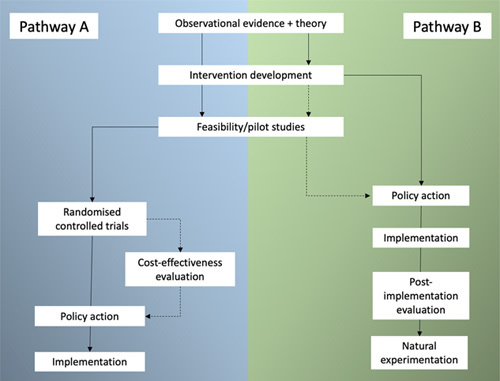
Two pathways to policy action (A) classic clinical pathway and (B) pragmatic public health pathway (adapted from Ogilvie et al^10^).

The challenges in using RCTs to evaluate complex interventions to overcome social determinants of health are well described,^8^,^9^ and most strategic decisions—particularly in Public Health—are made without the benefit of evidence from an RCT. So, what else constitutes acceptable evidence? Figure 1 (adapted from Ogilvie et al^10^) describes 2 pathways that lead to health policy change. The first (pathway A) includes RCTs and is more typical of the hospital-based system that is familiar to clinical hepatologists. Expert opinion and observational data are collected, collated, and presented. This leads to the development of an intervention, which is tested in an RCT and leads (usually with support from further trials, meta-analysis, and cost-effectiveness evaluation) to policy action. A recent example from clinical hepatology is the changing indications for carvedilol in patients with liver cirrhosis. Observational data indicated that beta-blockers should be effective at preventing decompensation in patients with clinically significant portal hypertension.^11^,^12^ These studies led to an RCT that showed positive results,^13^ and this has started to alter international policy.^14^

The second pathway (pathway B) is more typical of public health and will be less familiar to clinical hepatologists. Expert opinion and observational data lead to policy change, policy action, and the implementation of an intervention. A good example of a widespread practice in clinical hepatology that lacks evidence from RCTs (with the exception of a study in China^15^) is HCC surveillance with liver ultrasound. Observational data about the relative incidence of HCC in patients with liver cirrhosis and expert opinion have led to the practice being recommended in international guidelines.^16^,^17^ The impact of HCC surveillance has been evaluated in observational cohort studies that have compared outcomes for patients with HCC “exposed” to surveillance or presenting outside of surveillance.^18^ These studies are at risk of lead time bias and selection biases (including length-time bias) for which they have been partially adjusted.^18^ The results have been used to parameterize cost-effectiveness models and support the widespread implementation of surveillance.^19^ Despite the widespread implementation, some authors have advocated that there is still a need for an RCT, but others have highlighted the lack of acceptability, large sample sizes needed to demonstrate significant effects, and high study costs.^20^–^22^

In 2014, the Centre for Disease Control in the US recommended cohort screening for HCV of the baby-boomer generation.^23^ This was a massive program that received high-level criticism calling for an RCT.^24^ However, the call was met with a response from the clinical community that indicated such a trial was unacceptable. Through online responses, other experts cited the high costs involved, the timescale required, and that modeling had already explored some of the uncertainties that would be addressed by a trial. In a similar example, NHS England has recently funded a widespread scale-up of community testing for early-stage liver disease. The program follows the recent publication of the NHS long-term plan^25^ and a political focus on early identification of disease—specifically cancer. In keeping with pathway B in Figure 1, the policy has led to rapid implementation without utilizing the evidence-generation steps in pathway A.

What can help clinicians decide whether interventions implemented into practice without passing through the traditional hierarchy of medical evidence is the right thing for their patients and the communities they look after? As we have highlighted, observational data can help but are subject to biases that limit causal inferences. In the remainder of this article, we will discuss how natural experimental studies (henceforth abbreviated to NES)—sitting somewhere between experimental and observational research methods—can help. We describe this method in detail for the clinical audience of this journal because we believe NES are key to better evaluations of large-scale health interventions for patients at risk of, or with liver disease outside of the hospital walls. Unlike other research methods, they are undertaught and underutilized.

### What are NES?

To illustrate what we mean by NES, we will work through historical, famous, widely cited, but infrequently fully explained examples of Public Health research. It is well known that in 1854 John Snow identified the source of cholera outbreaks in London, UK, and undertook a simple Public Health intervention—he is famously credited with removing the handle from the Broad Street water pump—thereby cutting off a key source of contaminated water. However, the study design John Snow used to draw his conclusions is less well known.^26^

Sometime before his study, 1 of the 2 water companies serving London situated their intake pipe in the River Thames upstream of the city in (what turned out to be) less contaminated water. The other company continued to take water from the Thames as it ran through the city. To test his hypothesis that cholera was waterborne, John Snow looked at cholera cases in households served by each water company. He noted that the incidence of cholera in households served by the downstream water company was 10 times that of households served by the company with the upstream source. John Snow recognized the risk of bias and worked hard to prove that the supply of water to each household was not associated with other factors that could be associated with cholera (ie, confounders). In fact, he was able to show that the supply of water was almost random: many households were unaware of which water company they used, and neighboring houses were often served by different companies.

In his study, John Snow highlighted the “rules” that now define NES^10^,^27^ (Box 1) The “intervention” (in this case a change in water pipe location) should be outside of the researchers’ control, the allocation of the intervention should be “as if” random or at the very least variation in exposure should be unrelated to factors that may influence the outcome^7^,^28^,^29^ and the experiment should be relevant to current health policy/service decisions. Crucially, it should be possible for causal inferences to be drawn from the study.^30^ We will return to these rules again when we evaluate examples of NES in hepatology research.

Ground rules that define a natural experiment

1. Researchers lack control over the implementation of the intervention

2. Variation in exposure to the intervention should be unrelated to the outcome such that causal inference can be drawn

3. The intervention should be relevant to public health/health service decisions

**FIGURE 2:**
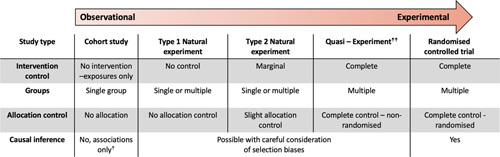
Observation to experimental design spectrum (adapted from Ogilvie et al.^10^).^†^This is not universally true, for example, the causal association between smoking and lung cancer is primarily based on observational data. For a different perspective on causality in observational research designs, see Vandenbroucke et al.^32^. Specific criteria that “upgrades” the strength of observational data such that causal inferences may be considered are available in the GRADE statement.^33^ ††The definition of quasi-experiment does vary—for an alternative, see de Vocht et al^29^.

Some authors have contended this relatively straightforward definition of NES, summarized by the Medical Research Council^30^ and Box 1, does not capture their full complexity. Dawson et al^31^ classify NES into type 1 and type 2 (Figure 2). Type 1 fits most closely with the MRC definition and the examples we have already discussed—researchers have no control over the implementation and exposure to the intervention. In type 2, researchers may have some control. For example, they could influence how and where a health intervention is being deployed to influence the seminatural formation of groups. Type 2 NES get close in structure to quasi-experimental designs, which are, in turn, closer to the RCT design (Figure 2). The term “quasi-experiment” is often used interchangeably with natural experiment, and there remains debate in the literature over their exact definitions.^29^ Generally, quasi-experiments are recognized to include designs where the researcher has full control of the intervention, but there is still an absence of control over randomization^31^ and hence would not meet the rules of the definition of NES (Box 1). A good example of a quasi-experimental study was when uptake of a researcher-led intervention relies on volunteers (forming the intervention “arm”) with people who do not volunteer to become a control group. In this example, very careful consideration needs to be given to controlling for potential confounders that are associated with the act of volunteering and the outcome of interest.^31^,^34^

NES have strengths over other study designs: they can evaluate the effect of events or interventions that are impossible to manipulate experimentally, interventions are generally less distorted than in strict experimental conditions, and control groups are less likely to alter their normal behaviors.^35^ In addition, NES can be used with retrospective data and are less susceptible to confounding than conventional observational designs.^29^ Accordingly, NES can provide strong causal information with large effect sizes^29^ that are comparable in some circumstances to randomized designs^36^ (Figure 2). However, to do this, NES need to be carefully planned, well conducted, and accurately reported.

### Examples of NES in hepatology

NES have been widely used in global health care–related research with a broad range of examples, including interventions aimed at reducing gun fatalities in the US,^37^ improving road safety,^38^,^39^ improving maternal health,^40^ reducing suicide with pesticides,^41^ and reducing cycling accidents.^42^ We will now consider a few examples of where NES have been used in studies relating to liver disease or the direct risks of liver disease (Table 1). In keeping with the recommendations in the recent European Association for the Study of the Liver–Lancet Commission^1^ and its previous editions,^47^ these studies have an appropriate focus on early identification or prevention of liver disease in community settings.

**TABLE 1:** Examples of natural experiments in hepatology

References	Aim of intervention	Intervention	Implementation of intervention	Group allocation process	Measured primary outcome	Method of analysis
Srivastava et al^43^	Reduce inappropriate referrals to hospital hepatology services	Referral pathway, including noninvasive fibrosis assessment before referral	Commissioned novel pathway in 2/25 CCGs referring patients to 3 hospital trusts	Iterative service development and funding in 2 CCGs (2—intervention) but not others (23—control)	Change in the proportion of patients referred to hospital care with significant fibrosis or cirrhosis	Compared OR of having primary outcome before and after pathway in intervention patients and between control and intervention patients after implementation
Hickman et al^44^	Reduce the prevalence of HCV in PWID	Multifaceted community HCV service innovation	Expanded HCV care pathways in 1 geographical area	Asymmetrical iterative service development with early adopter and late adopters	Chronic HCV prevalence in PWID	Adapted Bayesian synthetic control methods
Jugnariain et al^45^	Increase the proportion of PWID starting and completing treatment for HCV	Peers to support engagement with HCV treatment	Peer-supported engagement with HCV treatment	Asymmetrical iterative service development with early adopter and late adopters. Timing dependent on funding application and operational readiness	Total numbers starting treatment (presented as a relative ratio)	Mixed effects model
O’Donnell et al^46^	Reduce alcohol consumption	Minimum unit price for UK unit—50p (0.61$)	Implemented in Scotland	Policy implemented in Scotland, not implemented in England	No. grams of alcohol purchased per household	Interrupted controlled time series analysis

Abbreviations: CCGs, clinical commissioning groups; PWID, people who inject drugs.

Concerns about overburdening stretched hepatology services have led to novel pathway designs that stratify patients as “high risk” for significant liver disease before a referral is made (for an overview of novel pathways, see Abeysekera et al^48^). A good example is Srivastava et al^43^ published in 2019. This article has had impact with over 200 citations in 3 years. In the study, the authors compared the proportion of significant liver disease in patients referred to the hospital through a novel pathway with others that were referred without the novel pathway and showed that the pathway significantly reduced unnecessary referrals. The study broadly meets the “rules” for a NES (Box 1) (Table 1). The study met an important clinical/public health concern; the researchers lacked control over the implementation of the intervention, circumstance dictated which population was exposed, and there was a reasonable argument that the exposed and unexposed groups were broadly similar.

Our second and third examples describe interventions to enhance HCV treatment engagement in people who inject drugs (PWIDs). In both, the populations who are exposed to the intervention live in areas where there has been the early implementation of enhanced services for HCV treatment, and the “control” or unexposed populations live in areas with slow adoption of the interventions. Hickman and colleagues describe the study protocol for the Epitope study (results unpublished at the time of writing). They compare the prevalence of HCV in the Tayside area of Scotland to other parts of Scotland where HCV services for PWID were in their relative infancy.^44^ Jugnarain et al^45^ describe the impact of peer-supported engagement with HCV treatment in PWID living in areas of England where peer support has been implemented and compare the number starting and completing treatment with areas that have not started a peer-supported program. They observed a significant increase in the rates of treatment initiation and contended that this was unlikely to be due to hidden confounders:

“given the magnitude of the change and the large number of networks involved it is difficult to envisage a common confounding factor that could have led to the changes we observed.”

Our final example tested the impact of the implementation of the minimum unit alcohol pricing policy in Scotland. In many respects, this is a “classic” NES. Observational data^49^ describing the association between cost and consumption led directly to a policy change. Evaluation of the impact then relied on observational data and NES. O’Donnell et al^46^ compared the amount spent per household on alcohol in Scotland and England (where the policy was not implemented) and separately in northern areas of England—to control for “cross-border contamination.” The authors showed an immediate drop in alcohol purchasing in Scotland and no comparable decrease in England. The authors summarized the rationale and strength of their natural experiment:

“although the randomised controlled trial remains the ideal research standard, interrupted time series analysis provides a strong alternative where an experimental study design is infeasible or unethical, such as the evaluation of policy initiatives in healthcare.”

### Design and analysis in NES

**FIGURE 3:**
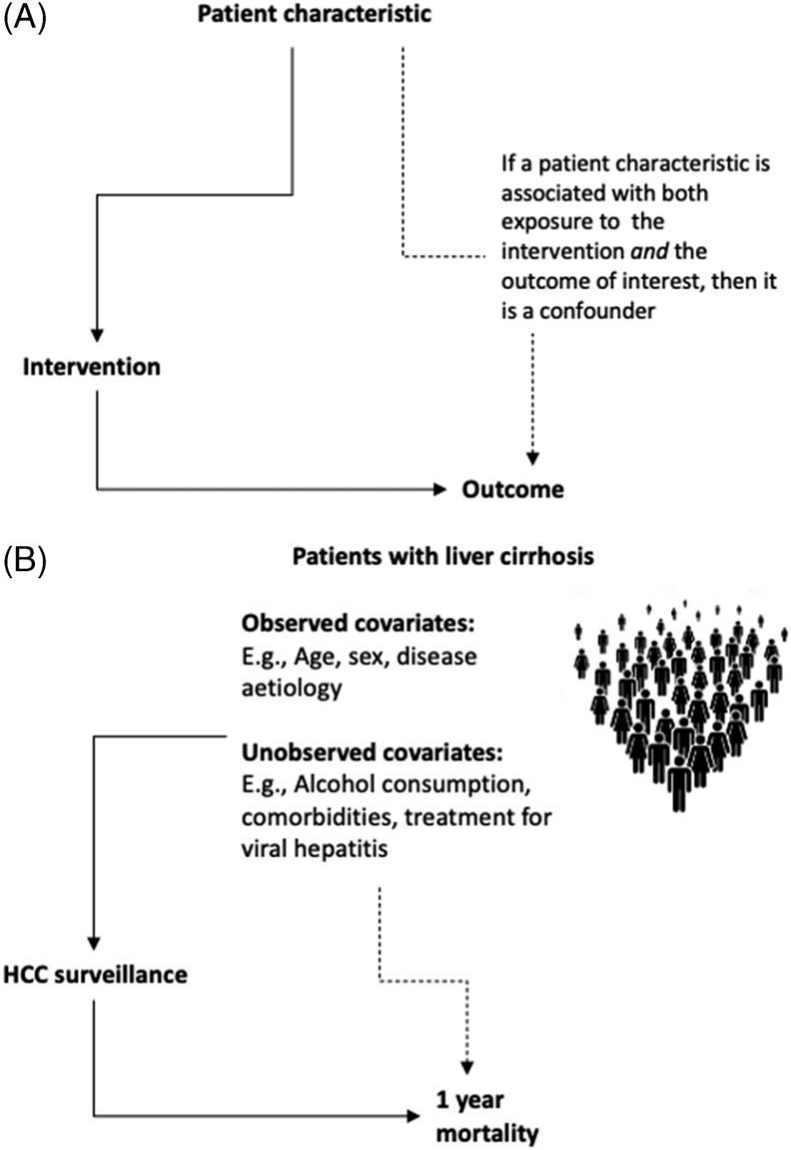
Confounding is visualized as a directed acyclic graph. In (A), as indicated by the arrows, a patient characteristic is associated with exposure to the intervention, and the outcome is therefore confounding. (B) Patient characteristics are associated with engagement with HCC surveillance, for example, abstinence from alcohol may plausibly be associated with increased attendance at ultrasound appointments and is plausibly independently associated with reduced 1-year mortality. When designing an observational study, it is important to measure the covariates that could introduce confounding and use design and analytical approaches that mitigate their impact. By design, natural experimental studies are devised *a priori* to deal with confounding and therefore strengthen causal inferences that can be drawn from the results.

By definition, in NES, the researcher has little or no influence over exposure to the intervention.^27^ In all NES, exposure to the intervention is therefore at risk of selection bias as the implementation is very rarely completely random—an exception may be a study that compares lottery winners to members of the general population. Selection bias becomes a problem when it leads to confounding. A confounder is a covariate associated with the intervention and the outcome of interest. Figure 3A illustrates this as a directed acyclic graph, and as an example, Figure 3B illustrates how observed and unobserved differences (covariates) between patients with cirrhosis exposed and unexposed to HCC surveillance could lead to confounding in observational studies evaluating its effectiveness.

The study design and analytical approach taken should be the best available to mitigate the effect of selection bias and confounding on the outcome. There are many approaches to maximize causal inferences in NES, which in many instances equally apply to observational and randomized designs. Broadly speaking, these approaches fall into 2 groups—those designed to deal with recorded covariates and those designed to deal with things the researcher does not know about the study population (see Figure 3B for an example). We summarize the approaches in Table 2 and highlight how our examples of NES in hepatology research have maximized causal inferences in the following text. A more comprehensive overview of different approaches to maximize causal inferences is available elsewhere.^30^,^50^

**TABLE 2:** Examples of study design and analytical tools to enhance causal inferences in natural experiments

	Positive effect	Limitation in NES
Tools to manage measured covariates as potential confounders		
Control population	Gives a counterfactual to support a more robust analysis	May not be available in natural experimental conditions. Likely to be unknown underlying differences between groups
Multiple control populations	Reduces confounding associated with just a single control group. Groups need to differ in a meaningful way such that potential confounders in 1 control group but not another can be dismissed	Challenging to identify more than 1 control group that closely matches the intervention group yet differ from each other
Matching, eg, through propensity scores	Creates a subpopulation with similar characteristics to those exposed to the intervention	Matching can only be ascribed using measured covariates
Regression analysis	Adjusts for observed differences between control and intervention groups	Cannot account for confounding caused by unobserved covariates
Tools to manage unmeasured covariates as potential confounders		
Repetition of experiment in multiple settings	Increases sample size, reduces unmeasured biases if factors associated with exposure to intervention differed between settings	May not be available, potentially costly
Mixed method design	Supports triangulation of qualitative and quantitative data	More costly and time consuming, nested qualitative study likely to need ethical approval
Difference in differences analysis	Follows the same unit through time and therefore is invulnerable to unobserved differences	Only applicable to data measured at 2 (or more) time intervals in the same unit or individual. Relies on underlying assumptions, eg, parallel trends assumption.
Time series analysis	Accounts for underlying trends in data before and after the implementation of the intervention	Needs data from multiple time points before and after the intervention is implemented. Time intervals need to be equal.
Instrumental variable (IV) analysis	Uses covariate associated with exposure to the intervention but not the outcome to control for unmeasured confounders	Difficult to identify IVs in NES that do not violate fundamental assumptions for their use

Abbreviation: NES, natural experimental studies.

Srivastava and colleagues compared patients referred through a novel service pathway to patients referred from other areas in London (UK), where the pathway had not been implemented. The results are presented as an “Odds” that patients seen in the clinic will have significant fibrosis/cirrhosis—that is, are they appropriate referrals? The results were positive with patients referred from General Practice (GP) with the novel pathway being more likely to have a significant disease; however, it is unlikely the patients coming from the 2 areas are exactly the same, that is, there will be some selection bias in exposure to the novel pathway. Had this same study been an RCT, the unit of randomization would have been GP practices. A confounder would therefore arise from a variable associated with GP services in one area that is associated with the outcome of interest (Figure 3). For example, an education program aimed at GPs in the intervention area could have improved the appropriateness of referral independently of the new pathway. To support their assertion that the new pathway (rather than hidden confounders) caused the improved selection of patients referred to secondary care, the authors conducted a supplementary analysis. Further analysis showed a significantly increased proportion of appropriate referrals within the intervention area if the novel pathway was followed compared with those where it was not.

**FIGURE 4:**
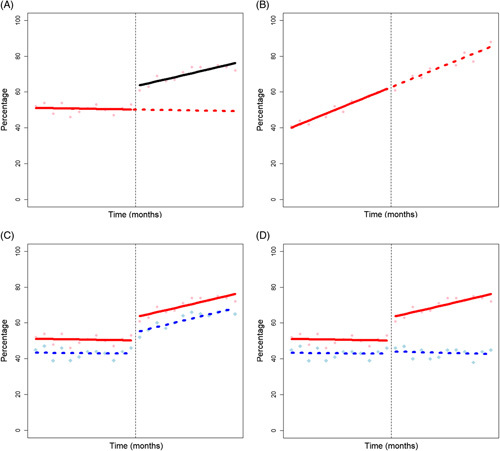
Graphical representation of interrupted time-series analysis (A&B) and a controlled interrupted time series analysis (C&D). Vertical black dotted lines represent the interruption - i.e. the implementation of the intervention. In A&B the solid red line represents the pre-intervention trend, black solid line the post intervention trend and the red dotted line represents the counterfactual (predicted trend). In C&D the red solid line shows the pre and post intervention trend in the group exposed to the intervention and the blue dotted line represents the pre and post intervention trends in the group exposed to control conditions.

However, in their analysis, Srivastava and colleagues do not account for background trends in the primary outcome. When outcomes are analyzed discreetly, underlying trends are unaccounted for, this can lead to misleading results. For example, the development of the intervention with community partners could have led to a change of behavior in referring primary care physicians before the novel pathway was introduced. The observed effect could have been a continuation of this behavior change after the pathway was introduced rather than an effect of the pathway itself. Figure 4A and B show 2 hypothetical time series data of time (*x*-axis) and a percentage (*y*-axis). In Figure 4A, the mean monthly percentage is 51% before the intervention versus 70% after. In Figure 4B, the means are 51% versus 74%, respectively. If just considering mean proportions before and after the intervention, we may determine that it was effective in both scenarios. However, the benefit of examining the trends in Figure 4 is clear—we can see the evidence of an intervention effect in Figure 4A and no effect in Figure 4B.

Interrupted time series (ITS) is a common analytical approach in NES (Table 2). A review in 2019 identified over 200 articles that reported using ITS in a health care setting (although only 116 met the full inclusion criteria for the review).^51^ As per our example, (Figure 4) in an ITS, equally spaced data points are compared before and after the intervention (the interruption) is implemented.^52^ To conduct ITS analysis, a large number (typically at least 8) of data points are needed before and after the interruption.^52^ Regression modeling is used to estimate the underlying trend in the preinterruption data and consequently the expected trend if the interruption had not occurred, what is termed the “counterfactual.”^53^ The counterfactual is a comparator for the observed postinterruption data to examine whether the interruption had an effect significantly different from the expected trend. In doing so, the ITS design controls for any pre-existing trends in the data.

However, ITS can still give misleading results: the before and after populations may not have the same characteristics, time may have affected the primary outcome independently of the tested intervention, and hidden environmental confounders that cannot be adjusted for may have altered the observed trends.

The addition of a group that is unexposed to the intervention adds validity by controlling for hidden confounding.^54^ O’Donnell et al^46^ (Table 1) conducted a controlled ITS. Two control groups were used, the whole of England and a sub-group that just included those in Northern England. Figure 4C and D illustrate the benefit of a control group using hypothetical data. Figure 4C illustrates similar effects in the control and intervention time series, indicating that a confounder—common to both groups—rather than the intervention is increasing the percentages. In Figure 4D, we see an absence of change in the control time series, supporting the assertion that the observed effect is a result of the intervention.

The control group needs to be carefully chosen. One needs to be confident that the control group is exposed to the same environmental influences as the intervention group—except for the intervention itself—and be confident that the control group cannot be affected by the intervention through contamination.

In their study protocol, Hickman and colleagues describe their intention to use an adapted causal impact synthetic control model^55^ to assess the impact of changing service design on HCV. The synthetic control population is based on preintervention population characteristics and provides a counterfactual trend against which the impact of the intervention can be compared. The use of a synthetic control population has the advantage of being less subjective and should ensure it is more representative of the wider population.^30^

### Conducting and reporting NES

One of our selected studies (Table 1) presents a protocol.^44^ The Medical Research Council (MRC) and others recommend the publication of a study protocol in advance of conducting NES. Otherwise, there is a risk of a blurring of intended target populations, outcomes, and analytical approaches.^9^,^56^ Alongside the robust approaches to assess causal inference we have described, a published *a priori* protocol adds validity to the findings and has the potential to broaden the acceptability of NES as admissible evidence for causation. For reference, a detailed framework of what to include in the protocol has been recently published.^29^

In their study, O’Donnell and colleagues used a recognized reporting guideline.^46^,^57^ The reporting guideline they used is specific to studies using an ITS design and describes 8 quality criteria. The first 4 criteria relate to the general quality of NES, and the remainder is specific to ITS. Alternatively, other authors recommend using the TREND guideline.^27^ These were developed by the Centre for Disease Control in the US to improve the quality of studies testing interventions designed to tackle the HIV epidemic and were modeled on the EQUATOR guidelines for RCTs.^58^ The TREND guidelines are now widely used, frequently requested by journal editors, and are specific for studies that evaluate interventions using nonrandomized designs.^59^ The TREND checklist includes 5 sections; many subsections are more applicable to quasi-experiments as they assume the researcher has control over the intervention and (nonrandomized) allocation of participants.^60^ The MRC gives an adapted, brief, and more specific summary of what should be reported in NES to convey validity (Table 3).

**TABLE 3:** Key information to report in natural experimental studies (NES)

Study component	Key information
Design	Describe the study design *a priori* in published protocol and in full study abstract and methods Describe how the design meets the definition for NES Report study using recognized reporting guidelines
Eligible and included population	Include a precise and detailed definition of the eligible population or service areas *a priori* in the published protocol and as part of the main manuscript Report the characteristics of included population and compare to control population (if using) Describe any selection biases in exposure to intervention Justify choice of control population Describe and justify the choice of measured covariates
Intervention	Define the intervention being tested Describe the level of researcher control over the intervention Describe the implementation landscape of the intervention Consider spillover effects between the intervention and control groups
Outcome and analysis	Define primary outcome and analysis plan *a priori* in published protocol and report clearly in study methods Describe and justify the use of design and analytic tools to maximize causal inferences Describe how residual confounding may bias the outcome

### Ethical considerations in NES

We argue that the use of NES in hepatology will help physicians adhere to the World Medical Association Declaration of Helsinki, specifically natural experiments will serve to enhance equity of access for disadvantaged and marginalized populations to health research and provide a means to test unproven interventions that have been implemented into practice.^61^ Other aspects of the declaration are also important when planning and conducting a natural experiment. Although the intervention is largely or totally outside of the researcher’s control, the physician-researcher still has obligations to prevent harm occurring to participants. This is more complex than in an RCT or quasi-experiment. Consider Jugnarain et al^45^ in Table 1. What if the peer-support program had been unexpectedly associated with reduced engagement with HCV treatment or the researchers observed unanticipated negative effects—so-called adventitious harms? The research team would have been ethically obliged to meet with commissioners, publish and publicize their findings and encourage consideration about the suspension of the service. However, the ability of a researcher to act to prevent harm in NES is usually limited. The analysis of a NES is typically conducted well after the intervention has been implemented (as in all of the examples we cite above)—therefore, the findings of the study cannot alter exposures that have already taken place.^31^

Research participants should always give informed consent^61^ for data collection and, in the case of RCTs and quasi-experiments, allocation/randomization to an intervention or control group. In NES, the intervention is outside of the researchers control so there is not a need to collect informed consent for this; however, ethical approval is still required for the collection and use of data about the participants unless it is aggregated, anonymized, and in public domain.^31^

### The future of NES in hepatology

In this review, we have described 2 pathways that lead to health policy action. One relies on the conventional hierarchy of evidence before the implementation of an intervention. The second relies on *post hoc* analysis. We have highlighted 3 examples of hepatology clinical practice that have followed this second pathway, including HCC surveillance program, baby-boomer screening for HCV, and a community program to identify compensated liver cirrhosis and advanced fibrosis. Importantly, these programs are being implemented alongside electronic health records and accessible “big data.”^62^ A reliance on conventional observational research designs to use this data and evaluate these programs has limitations. NES go some way to addressing these limitations, and we hope this article will provoke thought and debate about how they could be applied. Consider baby-boomer screening for HCV, which was recommended in 2012. Can NES address some of the concerns raised by Koretz et al^24^ about the effectiveness of the program? If the implementation of screening was asymmetrical (eg, between the US states), did naturally occurring exposed and unexposed populations take shape that is sufficiently similar and large enough to observe relative liver transplantation rates or death in the years that followed?

To address the upstream determinants of liver-related morbidity and mortality, the field of hepatology is moving toward a focus on large-scale public health interventions. Relatively cheap and safe interventions are being deployed in community settings. We argue NES are needed to test the effectiveness of these interventions, and the hepatology community needs to familiarize itself with their design, strengths, and limitations.
